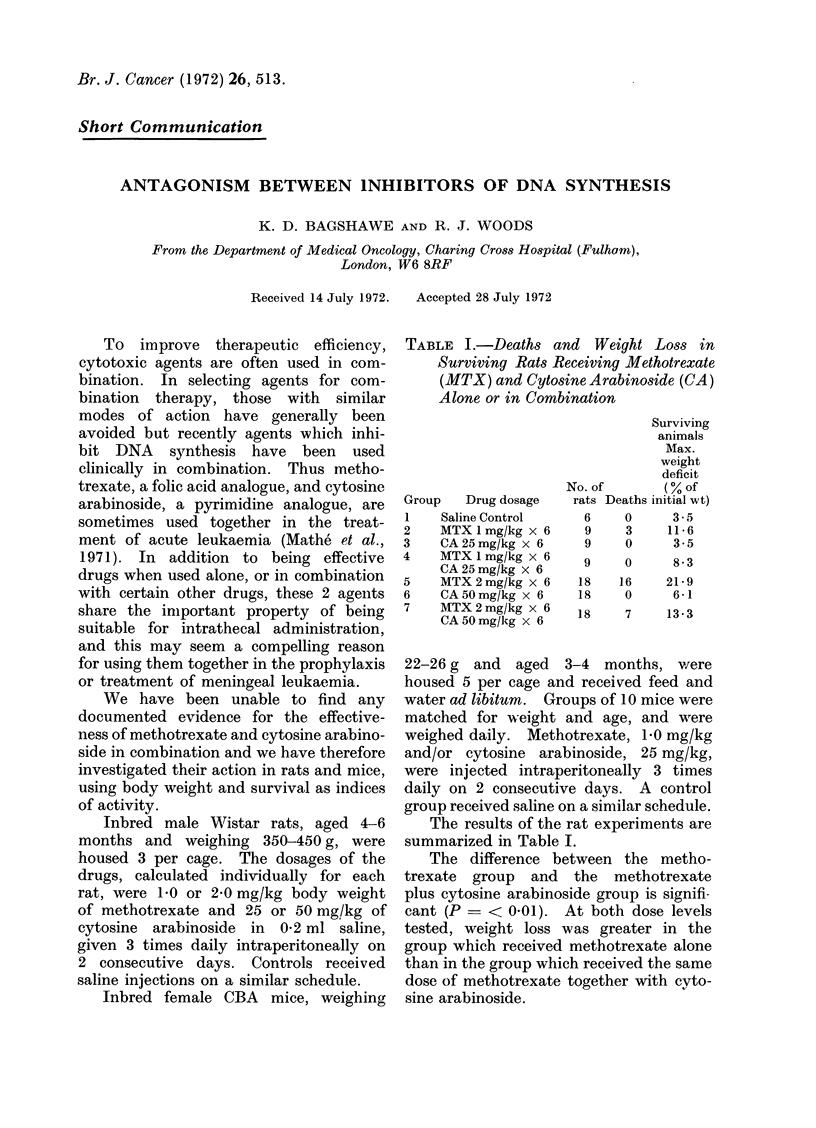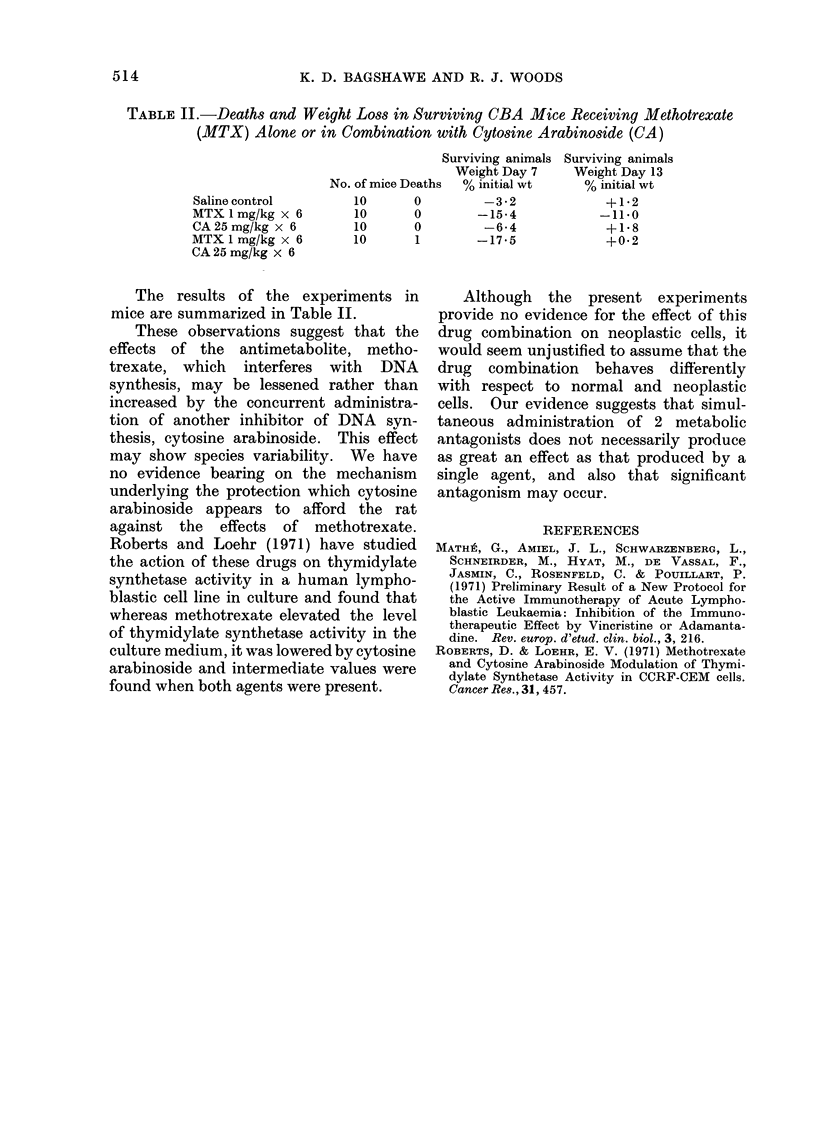# Antagonism between inhibitors of DNA synthesis.

**DOI:** 10.1038/bjc.1972.71

**Published:** 1972-12

**Authors:** K. D. Bagshawe, R. J. Woods


*Br. J. Cancer* (1972) **26**, 513.

***Short Communication***

### ANTAGONISM BETWEEN INHIBITORS OF DNA SYNTHESIS

K. D. BAGSHAWE AND R. J. WOODS

*From the Department of Medical Oncology, Charing Cross Hospital (Fulham), London, W6 8RF*

Received 14 July 1972. Accepted 28 July 1972

To improve therapeutic efficiency, cytotoxic agents are often used in combination. In selecting agents for combination therapy, those with similar modes of action have generally been avoided but recently agents which inhibit DNA synthesis have been used clinically in combination. Thus methotrexate, a folic acid analogue, and cytosine arabinoside, a pyrimidine analogue, are sometimes used together in the treatment of acute leukaemia (Mathé *et al.*, 1971). In addition to being effective drugs when used alone, or in combination with certain other drugs, these 2 agents share the important property of being suitable for intrathecal administration, and this may seem a compelling reason for using them together in the prophylaxis or treatment of meningeal leukaemia.

We have been unable to find any documented evidence for the effectiveness of methotrexate and cytosine arabinoside in combination and we have therefore investigated their action in rats and mice, using body weight and survival as indices of activity.

Inbred male Wistar rats, aged 4–6 months and weighing 350–450 g, were housed 3 per cage. The dosages of the drugs, calculated individually for each rat, were 1·0 or 2·0 mg/kg body weight of methotrexate and 25 or 50 mg/kg of cytosine arabinoside in 0·2 ml saline, given 3 times daily intraperitoneally on 2 consecutive days. Controls received saline injections on a similar schedule.

Inbred female CBA mice, weighing 22–26 g and aged 3–4 months, were housed 5 per cage and received feed and water *ad libitum*. Groups of 10 mice were matched for weight and age, and were weighed daily. Methotrexate, 1·0 mg/kg and/or cytosine arabinoside, 25 mg/kg, were injected intraperitoneally 3 times daily on 2 consecutive days. A control group received saline on a similar schedule.

TABLE I.—*Deaths and Weight Loss in Surviving Rats Receiving Methotrexate (MTX) and Cytosine Arabinoside (CA) Alone or in Combination*

| Group | Drug dosage | No. of rats | Deaths | Surviving animals Max. weight deficit (% of initial wt) |
|---|---|---|---|---|
| 1 | Saline Control | 6 | 0 | 3·5 |
| 2 | MTX 1 mg/kg × 6 | 9 | 3 | 11·6 |
| 3 | CA 25 mg/kg × 6 | 9 | 0 | 3·5 |
| 4 | MTX 1 mg/kg × 6<br>CA 25 mg/kg × 6 | 9 | 0 | 8·3 |
| 5 | MTX 2 mg/kg × 6 | 18 | 16 | 21·9 |
| 6 | CA 50 mg/kg × 6 | 18 | 0 | 6·1 |
| 7 | MTX 2 mg/kg × 6<br>CA 50 mg/kg × 6 | 18 | 7 | 13·3 |

The results of the rat experiments are summarized in Table I.

The difference between the methotrexate group and the methotrexate plus cytosine arabinoside group is significant ($P = < 0 \cdot 01$). At both dose levels tested, weight loss was greater in the group which received methotrexate alone than in the group which received the same dose of methotrexate together with cytosine arabinoside.

TABLE II.—*Deaths and Weight Loss in Surviving CBA Mice Receiving Methotrexate (MTX) Alone or in Combination with Cytosine Arabinoside (CA)*

| | No. of mice | Deaths | Surviving animals Weight Day 7 % initial wt | Surviving animals Weight Day 13 % initial wt |
|---|---|---|---|---|
| Saline control | 10 | 0 | −3·2 | +1·2 |
| MTX 1 mg/kg × 6 | 10 | 0 | −15·4 | −11·0 |
| CA 25 mg/kg × 6 | 10 | 0 | −6·4 | +1·8 |
| MTX 1 mg/kg × 6<br>CA 25 mg/kg × 6 | 10 | 1 | −17·5 | +0·2 |

The results of the experiments in mice are summarized in Table II.

These observations suggest that the effects of the antimetabolite, methotrexate, which interferes with DNA synthesis, may be lessened rather than increased by the concurrent administration of another inhibitor of DNA synthesis, cytosine arabinoside. This effect may show species variability. We have no evidence bearing on the mechanism underlying the protection which cytosine arabinoside appears to afford the rat against the effects of methotrexate. Roberts and Loehr (1971) have studied the action of these drugs on thymidylate synthetase activity in a human lymphoblastic cell line in culture and found that whereas methotrexate elevated the level of thymidylate synthetase activity in the culture medium, it was lowered by cytosine arabinoside and intermediate values were found when both agents were present.

Although the present experiments provide no evidence for the effect of this drug combination on neoplastic cells, it would seem unjustified to assume that the drug combination behaves differently with respect to normal and neoplastic cells. Our evidence suggests that simultaneous administration of 2 metabolic antagonists does not necessarily produce as great an effect as that produced by a single agent, and also that significant antagonism may occur.

### REFERENCES

MATHÉ, G., AMIEL, J. L., SCHWARZENBERG, L., SCHNEIRDER, M., HYAT, M., DE VASSAL, F., JASMIN, C., ROSENFELD, C. & POUILLART, P. (1971) Preliminary Result of a New Protocol for the Active Immunotherapy of Acute Lymphoblastic Leukaemia: Inhibition of the Immunotherapeutic Effect by Vincristine or Adamantadine. *Rev. europ. d'etud. clin. biol.*, **3**, 216.

ROBERTS, D. & LOEHR, E. V. (1971) Methotrexate and Cytosine Arabinoside Modulation of Thymidylate Synthetase Activity in CCRF-CEM cells. *Cancer Res.*, **31**, 457.